# A Hash-Based Quantum-Resistant Chameleon Signature Scheme

**DOI:** 10.3390/s21248417

**Published:** 2021-12-16

**Authors:** P. Thanalakshmi, R. Anitha, N. Anbazhagan, Woong Cho, Gyanendra Prasad Joshi, Eunmok Yang

**Affiliations:** 1 Department of Applied Mathematics and Computational Sciences, PSG College of Technology, Coimbatore 641004, India; ptl.amcs@psgtech.ac.in (P.T.); ani.amcs@psgtech.ac.in (R.A.); 2 Department of Mathematics, Alagappa University, Karaikudi 630004, India; anbazhagann@alagappauniversity.ac.in; 3 Department of Software Convergence, Daegu Catholic University, Gyeongsan 38430, Korea; wcho@cu.ac.kr; 4 Department of Computer Science and Engineering, Sejong University, Seoul 05006, Korea; joshi@sejong.ac.kr; 5 Department of Information Security, Cryptology and Mathematics, Kookmin University, Seoul 02707, Korea

**Keywords:** digital signature, chameleon signature, hash-based cryptography, homomorphic hash function, Preimage Resistance, key exposure free, random oracle model

## Abstract

As a standard digital signature may be verified by anybody, it is unsuitable for personal or economically sensitive applications. The chameleon signature system was presented by Krawczyk and Rabin as a solution to this problem. It is based on a hash then sign model. The chameleon hash function enables the trapdoor information holder to compute a message digest collision. The holder of a chameleon signature is the recipient of a chameleon signature. He could compute collision on the hash value using the trapdoor information. This keeps the recipient from disclosing his conviction to a third party and ensures the privacy of the signature. The majority of the extant chameleon signature methods are built on the computationally infeasible number theory problems, like integer factorization and discrete log. Unfortunately, the construction of quantum computers would be rendered insecure to those schemes. This creates a solid requirement for construct chameleon signatures for the quantum world. Hence, this paper proposes a novel quantum secure chameleon signature scheme based on hash functions. As a hash-based cryptosystem is an essential candidate of a post-quantum cryptosystem, the proposed hash-based chameleon signature scheme would be a promising alternative to the number of theoretic-based methods. Furthermore, the proposed method is key exposure-free and satisfies the security requirements such as semantic security, non-transferability, and unforgeability.

## 1. Introduction

An ordinary digital signature is not suitable for all applications that are personally or commercially sensitive. Consider the scenario where a software company issues a license of the software to the customer. When the company embeds an ordinary signature into its product to assure the customer Alice that the vendor provides the software, Alice can make several copies of the software and sell them at a lower price. She can prove that the software is from the vendor by presenting the vendor’s signature on the product. A privacy-protecting signature can be embedded in the product by protecting the company’s reputation and assuring the customer of having quality software. Though undeniable signature [1] proposed by Chaum et al. provides controlled dissemination of signature, a group of verifiers can verify it simultaneously, without telling the prover that he is proving the signature to too many people. This is done by setting the challenge collectively so that no subset of the verifiers could set the challenge on their own. To overcome the above-mentioned limitations of undeniable signature, Krawczyk & Rabin introduced chameleon signature [2], which prevents the dissemination of signed information by the recipient of the signature. It also preserves the non-repudiation property in the event of legal disputes.

Though undeniable signature and chameleon signature both provide non-transferability and non-repudiation, the former requires an interactive protocol between the signer and the verifier for the purpose of verifying the signature, which is based on zero-knowledge proof. It is quite natural that such additional properties and techniques increase the complexity of undeniable signature relative to ordinary signature in terms of computational and communication costs. As the chameleon signature scheme is non-interactive, it does not require the zero-knowledge paradigm, and hence its computational complexity is less compared to the undeniable signature scheme. Like an ordinary digital signature scheme, the chameleon signature scheme is constructed with the basic approach of the hash-and-sign paradigm. First, the message digest is computed using a special hash function called chameleon hash function, which uses the public key of the recipient of the signature. Like a cryptographic hash function, it provides collision resistance except for the trapdoor holder (recipient) of the public key. As a result, finding collisions to chameleon hash value is computationally infeasible even for the signer who produces the hash value, but it is feasible for the recipient to compute collisions using his trapdoor key. When a recipient receives a chameleon signature on a message, through collision finding, the recipient is able to produce different messages for the same signing value as the original one. Thus, the underlying chameleon hash function allows the recipient to forge the signature generated by the signer. The signatures generated by the signer and by the recipient are indistinguishable. This ability of the recipient to forge an indistinguishable signature prevents him from revealing the validity of the original signature made by the signer to a third party. Thus, the chameleon hash function is non-transferable. However, in the case of disputes, the signer can easily prove that the contested signature by the recipient is a forgery by producing a message–signature pair that has the same hash value as that of the contested message–signature pair. As the signer cannot compute a collision, the message–signature pair produced by the signer is taken as original, while the contested signature is considered forgery. The early designs of chameleon signature schemes are key-exposure, i.e., collisions that reveal the recipient’s secret key. Because the key is exposed, the recipient is unable to use the signature scheme. Calculating hash collisions, putting the principle of non-transferability to the test. As a result, the major key exposure concern, as well as its related issues such as revocation and redistribution of keys, must be addressed. Henceforth, a key-exposure-free chameleon hash is proposed in a graph setting. It uses a double trapdoor mechanism. The proposed chameleon hash system is used to create a chameleon signature scheme. The non-transferability of the signature thus produced is simply achieved from the underlying chameleon hash.

## 2. Related Work

Krawczyk & Rabin proposed the first chameleon signature scheme in [2]. It suffers from a key exposure problem which means that, when the recipient does forgery through collision computation, the signer can easily retrieve the recipient’s secret key. This creates a strong disincentive for the recipient to forge chameleon signatures which partially undermines the concept of non-transferability. Zhang et al. presented chameleon hash schemes in an ID-based setting in [3]. The proposed two schemes from bilinear pairings are not key-exposure-free. Ateniese & de Medeiros in [4] identified that the problem of key exposure on forgeries threatened the claim of non-transferability and provided a solution to the problem through an identity-based chameleon hash function. In their scheme, the signature forgery by the recipient results in the recovery of the recipient’s trapdoor information associated with that single transaction by the signer. However, in other transactions, the signer could not be able deny the signature on any message. Thus, the authors provide a solution but do not solve the problem of key exposure completely. To overcome this drawback, Chen et al. proposed the first complete key-exposure free chameleon hash function in the gap Diffie–Hellman group with bilinear pairings [5].

In [4], Ateniese & de Medeiros introduced three nonpairing based key exposure-free systems, two of which are based on Integer Factorisation Problem (IFP) and one on Diffie–Hellman and the Discrete Logarithm Problem (DLP). Gao et al. developed a factoring-based chameleon hash function [6] and demonstrated its security by using a modified Rabin signature technique. Later, Gao et al. introduced a DLP-based key-exposure free chameleon hash function [6]. They modified the basic construction of the chameleon hash method [2] to allow a multi-trapdoor by considering the hash algorithm as a one-round protocol that runs between the signer and the specified recipient. This architecture differs slightly from Krawczyk & Rabin’s original concept of chameleon hash. Later, Chen et al. suggested a DLP-based key-exposure-free chameleon hash and signature system that did not need the gap Diffie–Hellman groups [7]. Pan et al. proposed a family of chameleon hash functions and strongly unforgeable one-time signature schemes based on DLP over inner automorphism groups [8].

Integer factorization and discrete logarithm problems provide a secure basis for the above schemes and are threatened by quantum computers by Shor’s algorithm [9]. Therefore, it is necessary to come up with alternative schemes that resist quantum computer attacks. Although quantum computers are currently in their infancy [10,11], the ongoing development of quantum computers and their theoretical ability to compromise modern cryptographic schemes has prompted the development of post-quantum cryptographic schemes. A cryptographic technique connected with a computational problem that cannot be solved in polynomial time on a quantum computer is currently referred to as post-quantum security. This includes the fact that addressing the problem on a traditional computer is impossible. Because lattice-based signatures are thought to be secure against quantum computers, they have become fascinating alternatives to the current techniques, such as RSA, ECDSA, and others. Several lattice-based cryptographic signature techniques have been proposed since Ajtai’s original work [12]. In hard random lattices, Xie et al. suggested a homomorphic chameleon function based on the Small Integer Solution (SIS) problem [13].

Other promising post-quantum signature schemes are hash-based signature schemes. Hash functions are sufficient to have an efficient and a secure transmission of data. As hash functions are one-way, hash-based signatures provide certain advantages over digital signatures based on trapdoor functions. The following are the advantages of hash-based signatures: they require less security assumptions than number-theoretic signature schemes, they are very efficient, and their security is well understood. The generic attacks on hash function and pseudorandom generator determine the security parameter. The minimum security parameter recommended is determined by the security level of preimage and collision resistance of the hash function. When classical computers are used, then finding a preimage of the hash function $H:{\left\{0,1\right\}}^{2k}\to {\left\{0,1\right\}}^{k}$ by exhaustive search costs $2^{k/2}$ and finding a collision by birthday attack costs $2^{k/2}$. However, when quantum computers are used, the Grover algorithm [14] requires $2^{k/3}$ evaluations of the hash function to find a collision and requires $2^{k/3}$ searches to find a preimage of *H*. For a pseudorandom generator $PG:{\left\{0,1\right\}}^{k}\to {\left\{0,1\right\}}^{2k}$, the attacker tries to find the patterns in the inputs example by guessing the PG seed that ends up at a target value after a number of rounds of the chaining function. All generic attacks again cost $2^{k}$ in pre-quantum and $2^{k/2}$ in post-quantum.

Chen [15] proposed a PDP protocol which consists of an algebraic signature that uses a hash function with homomorphic property. It is shown that the homomorphic hash function enables fast and efficient retrieval of content and provides provable data possession and data integrity protection in cloud storage. Hence, this paper proposes a simple construction of a chameleon hash scheme under graph setting with minimal requirements such as homomorphic hash function and homomorphic pseudorandom generator and without complex algebraic computation. The security of the chameleon hash scheme relies on the Preimage Resistance (PR) of the hash function. The chameleon signature scheme constructed with the proposed chameleon hash scheme easily achieves the non-transferability property. In addition, the method obtains the unforgeability property directly from the unforgeability of the underlying signature scheme used.

The paper is organized as below: Section 3 contains a brief description of basic concepts of graph theory, the outline of a chameleon hash and signature scheme, and its security model. Section 4 proposes an efficient hash-based chameleon hash and signature scheme with DAG, and Section 5 presents an algorithm for the construction of a DAG. Section 6 presents the security analysis of the proposed method, and Section 7 discusses the performance analysis and comparison. Finally, Section 8 concludes the paper.

## 3. Preliminaries

In this section, few graph preliminaries [16] that are required for the proposed scheme and definitions and properties of chameleon hashing and signatures are briefed.

**Definition** **1.** ***Directed graph:*** *It is an ordered pair G=(V,E) where V is a finite set of elements called vertices and E is a set of ordered pairs of vertices called directed edges.*

**Definition** **2.** ***Degree:*** *In a directed graph, indegree of a vertex v is the number of edges (u,v)∈E which are pointing to the vertex v. Outdegree of a vertex v is the number of edges (v,u)∈E which are coming from the vertex v. The sum of indegree and outdegree is the total degree of v.*

In a directed graph, the vertices with indegree zero are called source vertices and the vertices with outdegree zero are called sink vertices. The vertices which are neither sources nor sinks are called internal vertices.

**Definition** **3.** ***Path:*** *In a directed graph, a path $\left(v_{0},v_{l}\right)$ is a sequence of vertices $\left(v_{0},\ldots ,v_{l}\right)$ such that $(v_{i-1},v_{i})\in E$, ∀i=1,…,l. Its length is the number of occurrences of edges in it.*

**Definition** **4.** ***Cycle:*** *In a directed graph, a cycle is a closed path in which all the vertices are distinct except that $v_{0}=v_{l}$.*

**Definition** **5.** ***DAG:*** *A directed acyclic graph is a directed graph with no cycles.*

For simplicity, only DAGs which have a single source *s* with outdegree 1, a single sink *t* with indegree 1, and n>0 internal vertices where each vertex that has a total degree of 3 is considered. Such graphs have only two types of internal vertices, namely expansion vertices and compression vertices. Expansion vertices are vertices with indegree 1 and outdegree 2, and compression vertices are those with indegree 2 and outdegree 1. A DAG *G* with *n* internal vertices where *n* is even will have n/2 expansion vertices and n/2 compression vertices. This particular type of DAG is considered throughout the paper.

**Definition** **6.** ***Cut;*** *In a DAG G=(V,E) with a source edge $e_{s}$ and a sink edge $e_{t}$, a cut is defined as a nontrivial partition $W=\left(U,V\setminus U\right)$ of the vertices such that $e_{s}$ is in the edge set of U and $e_{t}$ is in the edge set of V∖U. A cut is represented by a single set of vertices U with the convention that $e_{s}\in U$.*

**Definition** **7.** ***Cross edge:*** *A cross edge e=(u,v) of a cut $W=\left(U,V\setminus U\right)$ is defined as an edge in which u∈U and v∈V∖U. The set of edges crossing W is denoted as (W).*

**Definition** **8.** ***Poset;*** *A set P with a reflexive, antisymmetric and transitive relation ⪯ defined on it is a partially ordered set or poset denoted by (P,⪯).*

**Definition** **9.** ***Comparable:*** *Two elements a and b of a poset (P,⪯) are called comparable if either a⪯b or b⪯a. When neither a⪯b nor b⪯a, then the elements a and b are called incomparable.*

**Definition** **10.** ***Antichain:*** *A subset U⊆P is called an antichain if every pair of elements of U is incomparable. The maximal cardinality of antichains in P is called the width of a poset (P,⪯), and it is denoted by w(P).*

**Definition** **11.** ***Allowable set:*** *Let T be a set of edges in G. The set of directly computable nodes is defined as the set of nodes all of whose incoming arcs are in T. The set of computable nodes is defined recursively as the set of nodes computable from the union of T with the outgoing edges of the directly computable nodes. The set T is called an allowable set if it is verifiable and consistent. When T is allowable, the public key is obtained from the set of its computable nodes and the set either contains all the outgoing edges from a node or none from it.*

**Definition** **12.** ***Minimal allowable set:*** *An allowable set is called a minimal allowable set if it is not verifiable when any edge is omitted from the set.*

**Definition** **13.** ***Poset:*** *It is a set $\left(G^{*},⪯\right)$ that consists of minimal allowable sets of G with a partially ordered relation ⪯ defined on it. Two minimal allowable edge sets U and V of a poset are related as U⪯V if and only if the set of computable nodes of V is a subset of the set of computable nodes of U.*

**Definition** **14.** ***Comparable:*** *Two minimal allowable sets in G are said to be compatible if neither of the sets of computable nodes is a subset of the other. Then, the corresponding sets of computable nodes are incomparable elements in the associated poset.*

### 3.1. Chameleon Hash Scheme

A chameleon hash function is a collision-resistant trapdoor hash function linked to a public/secret key combination (pk,sk). Anyone with access to the public key pk can easily compute the hash value for each input. Except for the trapdoor key sk holder, no one can efficiently compute a collision on any given input.

The following three polynomial-time algorithms define the formal definition of a chameleon hash scheme [7]:  

**Key Generation**$\left(1^{k}\right)$: A probabilistic polynomial-time algorithm that generates a public/secret key pair (pk,sk) based on the security parameter *k*.
**Hashing Computation:** A probabilistic polynomial-time algorithm that computes and outputs the chameleon hash value $G_{pk}\left(M,r\right)$ given a public key pk, a message *M*, and a random parameter *r*.
**Collision Computation:** A deterministic polynomial-time method that outputs $r^{\prime }$ such that $G_{pk}\left(M,r\right)=z^{{}^{\prime }}=G_{pk}\left(M^{\prime },r^{\prime }\right)$ when given the secret key sk, a message *M*, a random parameter *r*, and another message $M^{\prime }$ as inputs.  

Furthermore, if *r* is taken from a uniform probability distribution, the distribution of $r^{\prime }$ is computationally indistinguishable from the uniform.

The following properties should be met by a secure chameleon hash technique [7]:  

**Collision resistance:** Without knowing the trapdoor key sk, there is no efficient algorithm that, given a message *M*, an auxiliary random parameter *r* and another message $M^{\prime }$, outputs $r^{\prime }$ with a non-zero probability that $G_{pk}\left(M,r\right)=z^{{}^{\prime }}=G_{pk}\left(M^{\prime },r^{\prime }\right)$.
**Semantic security:** Let $E\left[X\right]$ denote the entropy of a random variable *X*, and  $E\left[X|Y\right]$ denote the entropy of a random function *Y* of *X* given the value of the variable *X*. If the conditional entropy $E\left[M|z^{\prime }\right]$ of a message given its chameleon hash value $z^{\prime }$ equals the total entropy $E\left[M\right]$ of the message space, the system is semantically secure.
**key-exposure freeness:** If a recipient has never computed a collision on a chameleon hash $G_{pk}\left(M,r\right)$, no adversary will be able to identify an efficient algorithm to compute a collision on that $G_{pk}\left(M,r\right)$. This is true even if the adversary has access to the collision computation oracle for polynomially many queries on tuples $\left(M_{i},r_{i}\right)$ of his choosing, except for the challenge query.

### 3.2. Chameleon Signature Schemes

A message’s chameleon signature is created by digitally signing the message’s chameleon hash value. A chameleon signature scheme, as defined by Chen et al., consists of the techniques described in [7]:  

**Key Generation**$\left(1^{k}\right)$: A probabilistic polynomial-time algorithm that outputs a public/secret key pair $\left(pk_{S},sk_{S}\right)$ for the signer and $\left(pk_{V},sk_{V}\right)$ for the verifier based on the security parameter *k*.
**Sign:** A probabilistic polynomial-time algorithm that outputs a signature σ on the chameleon hash value $z^{{}^{\prime }}$ using the recipient’s public key $pk_{V}$, the signer’s secret key $sk_{S}$, a random string *r*, and a message *M* as inputs.
**Verify:** A deterministic polynomial-time procedure that outputs Accept if σ is valid else outputs Reject given the the recipient’s public key $pk_{V}$, the signer’s public key $pk_{S}$, the random string *r*, the message *M*, and the signature σ.
**Denial protocol:** A protocol between the signer and the judge that is not interactive. The signer submits a valid collision $\left(M^{\prime },r^{\prime }\right)$ to the judge when given a chameleon signature σ on a message *M*. If $M\neq M^{\prime }$ and σ are both legitimate, the judge concludes that the signature σ on the message *M* is forged.

### 3.3. Security Requirements of Chameleon Signature Schemes

The following are the properties of a chameleon signature scheme: unforgeability, non-transferability, non-repudiation, and deniability:  

**Unforgeability:** A valid chameleon signature cannot be generated by a third party. Furthermore, a recipient can only produce a forgery for a chameleon signature that was previously generated by the signer.
**Non-transferability:** Because the recipient can forge a signature, the recipient cannot persuade a third party that the signer generated a signature on a specific message.
**Non-repudiation:** The signer is not allowed to deny legitimate signature claims.
**Deniability:** By providing a collision to the chameleon hash value, the signer can deny a forgery of the signature.

## 4. Proposed Hash-Based Chameleon Hashing and Signature Scheme

In this section, first, a chameleon hash function is constructed, and then a signature scheme is designed using the proposed hash function.

### 4.1. Construction of a Chameleon Hash Scheme

The proposed chameleon hash scheme consists of the following polynomial-time algorithms:  

**Key Generation**$\left(1^{k}\right)$: Let *G* be a publicly known DAG. Associate a homomorphic hash function $H\;:\;F_{2}^{k}\;\times \;F_{2}^{k}\;\to \;F_{2}^{k}$ in the compression vertices and a homomorphic pseudorandom generator $PG:F_{2}^{k}\to F_{2}^{k}\times F_{2}^{k}$ in the expansion vertices of *G*. The public parameters are *G*, *H* and PG with respect to the security parameter κ. The algorithm randomly chooses $sk\in F_{2}^{k}$ and computes $pk=Ext_{\left\{e_{t}\right\}}\left[sk:G(e_{s})\right]$. It chooses a cut *W* in *G*. Let (W) be the cross edges of the cut *W* with |W|=l. It chooses a labeling $r=(r_{1},r_{2},\ldots ,r_{l})$ for (W) where $r_{i}\in F_{2}^{k}$ is the labeling of the *i*’th edge in (W) and computes $r^{{}^{\prime }}=Ext_{\left\{e_{t}\right\}}\left[r:G(W)\right]$. It outputs $(pk,\left(\left(W\right),r^{{}^{\prime }}\right)$ as a public key, where pk is the permanent public key and $\left(\left(W\right),r^{{}^{\prime }}\right)$ is the ephemeral public key which is changed periodically. The corresponding private key is (sk,r).

**Hashing Computation:** On input $\left(M,pk,\left(W\right),r^{{}^{\prime }}\right)$, the algorithm computes

$h^{{}^{\prime }}=Ext_{\left\{e_{t}\right\}}\left[h(M):G(W)\right]$

$G_{pk}\left(M,r^{{}^{\prime }}\right)=h^{{}^{\prime }}⊕r^{{}^{\prime }}⊕pk=z^{{}^{\prime }}$

Outputs chameleon hash $z^{\prime }$, for the tuple $\left(M,r^{\prime }\right)$.

**Collision Computation** For any valid hash value $z^{\prime }$, the collision computation algorithm computes a hash collision with the trapdoor key (sk,r) as follows: $z=h\left(M\right)⊕r⊕Ext_{\left\{(W)\right\}}\left[sk:G(e_{s})\right]$

$r^{*}=z⊕h\left(M^{*}\right)⊕Ext_{\left\{(W)\right\}}\left[sk:G(e_{s})\right]$.

$r^{{*}^{\prime }}=Ext_{\left\{e_{t}\right\}}\left[r^{*}:G\left(W\right)\right]$

The algorithm outputs a collision tuple $\left(M^{*},r^{{*}^{\prime }}\right)$ for the chameleon hash value $z^{{}^{\prime }}$.

### 4.2. Construction of the Chameleon Signature Scheme

A chameleon signature scheme is constructed by having the proposed chameleon hash function in a standard signature scheme of the same public key setting. The public key and the secret key of the proposed chameleon hash scheme are related in terms of a DAG. Hence, it is convenient to design a chameleon signature scheme by combining the proposed chameleon hash scheme and a graph-based signature scheme.

In the proposed signature scheme, each of the signer *S* and verifier *V* chooses a DAG and a secret value and labels the source edge of their DAG with the secret value and labels all the other edges by means of extension labeling from the source edge. The label obtained at the sink edge is their public key. The signer first computes the chameleon hash value for a message using the verifier’s DAG $G_{V}$ and his public key. Then, he computes all minimal allowable sets for the graph $G_{S}$ and defines a partially ordered relation ⪯ on it. The associated poset of $G_{S}$ is $\left(G_{S}^{*},⪯\right)$ with width $w(G_{S}^{*})$. Then, with an efficient mapping function, the signer maps the chameleon hash value to a minimum allowable set in the antichain of width $w(G_{S}^{*})$. Then, he outputs the values associated with the minimal allowable set with respect to his secret key as the signature for the chameleon hash value.

Signatures thus obtained are verifiable with respect to the signer’s public key and are compatible which means that without inverting hash functions or pseudorandom functions, no signature can be computable from the signatures of different messages. Consequently, the essential requirements for the proposed signature scheme are maximal antichain of minimal allowable sets and an efficiently computable collision-resistant mapping from the hash space to the maximal antichain. In addition, for the chameleon signature scheme to be recipient-specific, the hash computation has to be done on the verifier’s graph, and signature computation has to be done on the signer’s graph.

The following algorithms describe the proposed Hash-Based Chameleon Signature (HBCS) scheme:  

**Key Generation $\left(1^{k}\right)$:** The signer *S* and the verifier *V* selects DAG’s $G_{i}$(i=S,V). Let *H* be a homomorphic hash function and PG be a homomorphic pseudorandom generator associated with the compression vertices and expansion vertices of $G_{i}$(i=S,V). The signer randomly chooses $sk_{S}\in F_{2}^{k}$, computes $pk_{S}=Ext_{\left\{e_{t}\right\}}\left[sk_{S}:G_{S}\left(e_{s}\right)\right]$ and outputs the public key as $\left(pk_{S},\left(\left(U\right),t^{{}^{\prime }}\right)\right)$ where $pk_{S}$ is the permanent public key and $\left(\left(U\right),t^{{}^{\prime }}\right)$ is the ephemeral public key. The corresponding private key is $\left(sk_{S},t\right)$. Similarly, the verifier on his graph $G_{V}$, computes and outputs the public key $\left(pk_{V},\left(\left(W\right),r^{{}^{\prime }}\right)\right)$ where $pk_{V}$ is the permanent public key and $\left(\left(W\right),r^{{}^{\prime }}\right)$ is the ephemeral public key. The corresponding private key is $\left(sk_{V},r\right)$. Let $\mu :{\left\{0,1\right\}}^{k}\to G_{S}^{*}$ define a mapping from the chameleon hash space ${\left\{0,1\right\}}^{k}$ to the maximal antichain of $G_{S}^{*}$.
**Sign $\left(sk_{S},M\right)$:** Let $M\in {\left\{0,1\right\}}^{*}$ be the message to be signed. With the verifier’s secondary key $\left(\left(W\right),r^{{}^{\prime }}\right)$, the signer computes the chameleon hash value $G_{pk_{V}}\left(M,r^{{}^{\prime }}\right)=z^{{}^{\prime }}$ and maps $z^{{}^{\prime }}$ to $\mu (z^{{}^{\prime }})$ in the maximal antichain of $G_{S}^{*}$. Then, computes

$\lambda =Ext_{\left\{\mu (z^{{}^{\prime }})\right\}}\left[sk_{S}:G_{S}\left(e_{s}\right)\right]$

  The signature on *M* is $\sigma =(r^{{}^{\prime }},\lambda )$.
**Verify $\left(pk_{S},M,\lambda \right)$:** The verifier computes

$G_{pk_{V}}\left(M,r^{{}^{\prime }}\right)=z^{{}^{\prime }}$

  Verifies $pk_{S}=Ext_{\left\{e_{t}\right\}}\left[\lambda :G_{S}\left(\mu \left(z^{{}^{\prime }}\right)\right)\right]$
**Correctness**
$$\begin{array}{c} \begin{array}{cc} pk_{S} & =Ext_{\left\{e_{t}\right\}}\left[sk_{S}:G_{S}\left(e_{s}\right))\right]\\ \; & =Ext_{\left\{e_{t}\right\}}\left[Ext_{\left\{\mu (z^{{}^{\prime }})\right\}}\left[sk_{S}:G_{S}\left(e_{s}\right)\right]:G_{S}\left(\mu \left(z^{{}^{\prime }}\right)\right)\right]\\ \; & =Ext_{\left\{e_{t}\right\}}\left[\lambda :G_{S}\left(\mu \left(z^{{}^{\prime }}\right)\right)\right] \end{array} \end{array}$$
**Denial Protocol:** In the event of a legal dispute between the recipient and the signer, i.e., when the recipient submits a message–signature tuple $\left(M^{*},\left(r^{{*}^{\prime }},\lambda \right)\right)$ to the judge *J* and claims that it was generated by the signer, the judge first applies the above signature verification process and determines whether $\left(r^{{*}^{\prime }},\lambda \right)$ is a proper signature on $M^{*}$. If the verification fails, the judge rejects the alleged signature. Otherwise, he requests that the signer denies or accepts the claim. If the signer wishes to accept the signature, he simply informs the judge. If the signer wishes to declare the signature to be a forgery, he must provide a collision tuple $\left(M,\left(r^{{}^{\prime }},\lambda \right)\right)$ i.e., $G_{pk_{V}}\left(M,r^{{}^{\prime }},\lambda \right)=G_{pk_{V}}\left(M^{*},r^{{*}^{\prime }},\lambda \right)$. It is worth noting that, if $\left(M^{*},\left(r^{{*}^{\prime }},\lambda \right)\right)$ is a forgery, the signer can always supply the original message–signature tuple $\left(M,\left(r^{{}^{\prime }},\lambda \right)\right)$ that differs from $\left(M^{*},\left(r^{{*}^{\prime }},\lambda \right)\right)$. If $\left(M^{*},\left(r^{{*}^{\prime }},\lambda \right)\right)$ is valid, the signer cannot provide a collision on the hash value without knowing the secret key of the recipient. As providing collision is equivalent to finding the preimage of the hash function, which is shown in Theorem 1, the signer cannot provide collision and repudiate a valid signature. The inability of the signer in providing a collision on the chameleon hash value enables the judge to determine the signature is valid or forged.

## 5. Construction of DAG

Algorithm 1 is proposed to generate a DAG as explained in Section 3.
**Algorithm 1** DAG Construction1:let edge[n][n] be the matrix denoting the edges and initialized to 0.2:edge[1][2]←13:edge[n−1][n]←14:**for**i←1 to n−2 **do**5:      edge[i][i+2]←16:**end for**7:k←n/2                  ▹ Setting second edges for expansion vertices8:**if***k* is even **then**9:      **for** i←2 to *k* **do**10:          edge[i][k+i−1]←111:     **end for**12:**else if***k* is odd **then**13:      **for** i←2 to *k* **do**14:            **if** *i* is even **then**15:          edge[i][k+i]←116:            **else if** *i* is odd **then**17:          edge[i][k+i−2]←118:            **end if**19:      **end for**20:**end if**

**Figure 1:**
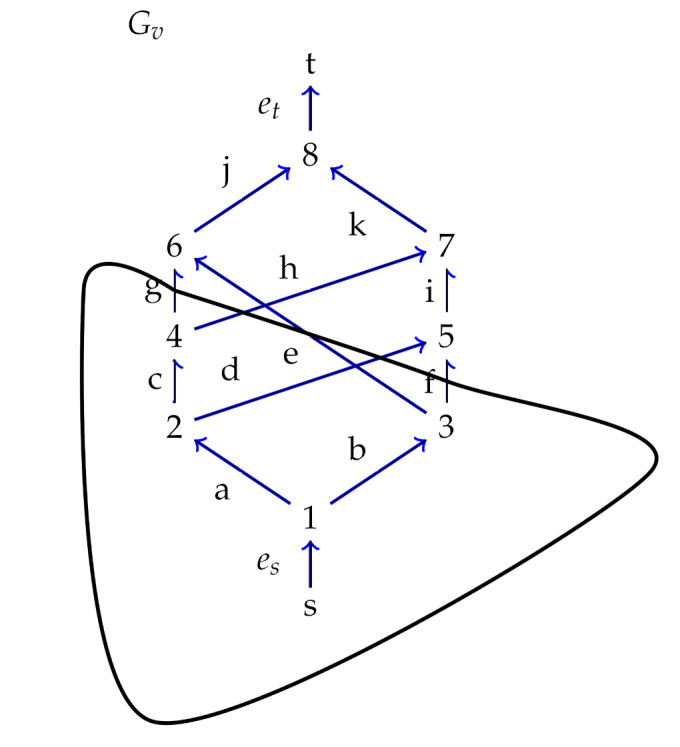
A DAG with a vertex cut.

**Figure 2:**
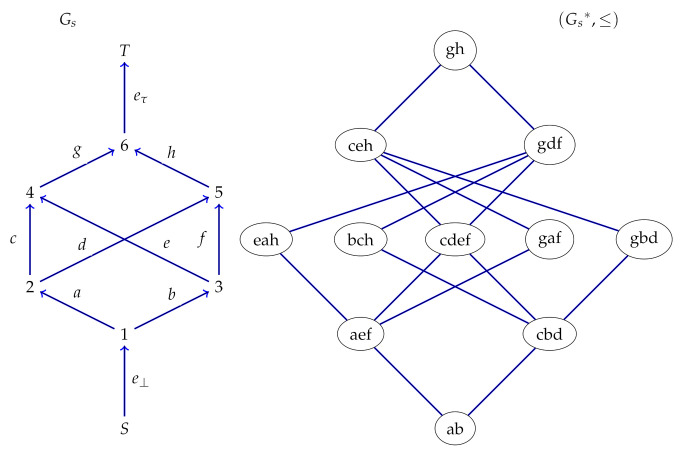
A DAG and its Poset.

DAG’s $G_{V}$ and $G_{S}$ are constructed and shown in Figure 1 and Figure 2, respectively.

In order to demonstrate the idea of the scheme, $G_{V}$ and $G_{S}$ are considered as the public graphs of verifier and signer, respectively. The hash function and pseudorandom generator are associated with the compression and expansion vertices of DAG’s respectively. The signer chooses his secret key $sk_{S}$ and computes the public key $pk_{S}$ using $G_{S}$. Similarly, the verifier chooses $sk_{V}$ and computes the public key $pk_{V}$ using $G_{V}$. In addition, the verifier chooses a cut *W* in $G_{V}$ and chooses random value *r* along the cross edges {g,h,e,d,f} of the cut and computes $r^{{}^{\prime }}=Ext_{\left\{e_{t}\right\}}\left[r:G_{V}\left(W\right)\right]$. The verifier publishes $\left(pk_{V},\left(\left(W\right),r^{{}^{\prime }}\right)\right)$ as public key. The signer computes the chameleon hash for h(M) in $G_{V}$ as $z^{{}^{\prime }}=\left(Ext_{\left\{e_{t}\right\}}\left[h\left(M\right):G_{V}\left(W\right)\right]⊕r^{{}^{\prime }}⊕pk_{V}\right)$. He computes the associated poset $G_{S}^{*}$ for $G_{S}$ as shown in Figure 2. As the width of $G_{S}^{*}$ is 5, the hash space consisting of at most 5 hash values can be mapped to the antichain of width 5. The elements in this antichain {e,a,h}, {b,c,h}, {c,d,e,f}, {g,a,f} and {g,b,d} are signature patterns. Suppose that the hash value $z^{{}^{\prime }}$ is mapped to an element {g,a,f}, then the signature is the value of $r^{{}^{\prime }}$ and the values of the edges {g,a,f} with respect to the signer’s secret key.

## 6. Security Analysis

This section examines the proposed chameleon hash function’s security properties. It also discusses the proposed chameleon signature scheme’s security against forgery, transferability, repudiation, and undeniability.

### 6.1. Security Analysis of the Proposed Hash-Based Chameleon Hash Scheme

In this subsection, the three properties of the chameleon hash scheme—collision resistance, semantic security, and key-exposure freeness—are proved.

**Theorem** **1.** *The proposed chameleon hash scheme is collision resistant provided the hash function is preimage resistant.*

**Proof.**  Let *A* be an adversary against collision resistance of chameleon hash function. Assume that *A* outputs collision tuples $\left(M,r^{\prime }\right)$ and $\left(M^{*},r^{{*}^{\prime }}\right)$.

Therefore,

$G_{pk_{V}}\left(M,r^{{}^{\prime }}\right)=G_{pk_{V}}\left(M^{*},r^{{*}^{\prime }}\right)=z^{{}^{\prime }}$

$\Rightarrow h^{{}^{\prime }}⊕r^{{}^{\prime }}⊕pk_{V}=h^{{*}^{\prime }}⊕r^{{*}^{\prime }}⊕pk_{V}=z^{{}^{\prime }}$

$\Rightarrow h^{{}^{\prime }}⊕r^{{}^{\prime }}=h^{{*}^{\prime }}⊕r^{{*}^{\prime }}$

$\Rightarrow h^{{}^{\prime }}⊕h^{{*}^{\prime }}=r^{{}^{\prime }}⊕r^{{*}^{\prime }}$

$\Rightarrow Ext_{\left\{e_{t}\right\}}\left[h\left(M\right):G_{V}\left(W\right)\right]⊕Ext_{\left\{e_{t}\right\}}\left[h\left(M^{*}\right):G_{V}\left(W\right)\right]=r^{{}^{\prime }}⊕r^{{*}^{\prime }}$

$\Rightarrow Ext_{\left\{e_{t}\right\}}\left[h\left(M\right)⊕h\left(M^{*}\right):G_{V}\left(W\right)\right]=r^{{}^{\prime }}⊕r^{{*}^{\prime }}$

$\Rightarrow (h\left(M\right)⊕h\left(M^{*}\right))$ is the trapdoor value of $\left(r^{{}^{\prime }}⊕r^{{*}^{\prime }}\right)$ along the cross edges of the cut *W* in $G_{V}$. Hence, by means of the consistent extension of $\left(h\left(M\right)⊕h\left(M^{*}\right)\right)$, the preimage of $\left(r^{{}^{\prime }}⊕r^{{*}^{\prime }}\right)$ under *H* is obtained. As finding the preimage of hash function is computationally infeasible, it can be concluded that, without the knowledge of the trapdoor, providing chameleon hash collision is hard. □

**Theorem** **2.** *The proposed chameleon hash scheme is semantically secure.*

**Proof.**  The computation of chameleon hash is $z^{{}^{\prime }}=Ext_{\left\{e_{t}\right\}}\left[z:G_{V}\left(W\right)\right]$ which equals $Ext_{\left\{e_{t}\right\}}\left[\left(h\left(M\right)⊕r⊕Ext_{\left\{(W)\right\}}\left[sk:G(e_{s})\right]\right):G_{V}\left(W\right)\right]$, where the value of *r* is chosen completely independent of *M*. In addition, the equation $\;z\;=\;h\left(M\right)\;⊕\;r\;⊕\;Ext_{\left\{(W)\right\}}\left[sk:G(e_{s})\right]$ implies that, for each fixed *M*, there is a one-to-one correspondence between the values of *z* and *r*.

Hence, $Pr(M|z^{{}^{\prime }})$= Pr(M|z) = Pr(M|r) = Pr(M). To prove the semantic security of the scheme, it is required to prove the conditional entropy $E\left[M|z^{{}^{\prime }}\right]=E\left[M\right]$.

$$\begin{array}{c} \begin{array}{cc} E[M|z^{{}^{\prime }}] & =-\sum_{M}\sum_{z^{{}^{\prime }}}Pr\left(M,z^{{}^{\prime }}\right)log\left(Pr\left(M|z^{{}^{\prime }}\right)\right)\\ \; & =-\sum_{M}\sum_{z^{{}^{\prime }}}Pr\left(M,z^{{}^{\prime }}\right)log\left(Pr\left(M\right)\right)\\ \; & =-\sum_{M}log\left(Pr\left(M\right)\right)\sum_{z^{{}^{\prime }}}Pr\left(M,z^{{}^{\prime }}\right)\\ \; & =-\sum_{M}log\left(Pr\left(M\right)\right)Pr\left(M\right)=E\left[M\right]. \end{array} \end{array}$$

Because the conditional entropy $E[\left(M|z^{{}^{\prime }}\right]$ equals the total entropy E[M], the chameleon hash value $z^{{}^{\prime }}$ reveals no information about *M*. As a result, the chameleon hash technique suggested is semantically secure. □

**Theorem** **3.** *The proposed chameleon hash technique does not require any key disclosure.*

**Proof.**  Even if the adversary *A* has been given oracle access to collision computation and has been given polynomially many queries with the tuples $\left(M_{i},r_{i}^{{}^{\prime }}\right)$, i≥1, of his choice, except for the challenge question, we assert that he cannot detect a collision on the hash value $z^{{}^{\prime }}=G_{pk}\left(M,r^{{}^{\prime }}\right)$ using an efficient technique. The adversary can use two sorts of attacks to find collision on $z^{{}^{\prime }}$. He might try to recover the secret values of $r^{{}^{\prime }}$ and pk along the cut’s cross edges in the first attack, and he might try to discover collision on $z^{{}^{\prime }}$ without recovering the secret keys in the second attack. The hash function’s Preimage Resistance (PR) is the target of the first attack. The second approach is to identify a collision on the given challenge $z^{{}^{\prime }}$ without knowing the private key, which is similar to cracking the graph-based one-time signature technique [16]. Because the graph-based one-time signature technique is safe against existential forgery in the standard model under a chosen message attack, it can be deduced that, even if the adversary has a polynomial number of signatures on messages, finding a collision on the hash $G_{pk}\left(M,r^{{}^{\prime }}\right)$ is difficult. As a result, the suggested chameleon hash has no key exposure. □

### 6.2. Security Analysis of the Proposed Hash-Based Chameleon Signature Scheme

In this subsection, the properties of the chameleon signature scheme: unforgeability, non-transferability, non-repudiation, and deniability are proved.

**Theorem** **4.** *Let G(V,E) be a directed acyclic graph with |V|=n, PG be a homomorphic pseudorandom generator and H be a homomorphic hash function. Let A be an adversary who performs an existential forgery under a chosen message attack against the proposed HBCS scheme with success probability ϵ. Then, $\epsilon \leq \left(\alpha \epsilon_{D}+\epsilon_{C}+\epsilon_{PG}+\epsilon_{H}\right)n+\epsilon_{G}$ where α≤n is the average number of predecessors of a random vertex in the graph and $\epsilon_{PG}$, $\epsilon_{H}$, $\epsilon_{C}$, $\epsilon_{D}$, and $\epsilon_{G}$ are, respectively, the success probabilities on inverting PG, inverting function H, finding collision on H, distinguishing random strings and pseudorandom strings apart, and finding collision on a chameleon hash function.*

**Proof.**  As HBCS is based on the hash-and-sign approach, the adversary can produce forgery either by finding a valid collision on the chameleon hash or breaking the underlying signature. By Theorem 1, the signer cannot produce a valid collision on a chameleon hash value. The construction of the proposed underlying signature is based on the signature schemes proposed by Hevia et al. in [16] and Dod et al. in [17]. Hence, the security proof of the underlying signature scheme follows their proof. The proof is given by the following games between the adversary *A* against the signature and Challenger *C*. Each game is a slight modification of the preceding game in a way that the difference between the two games can be evaluated. Thus, the quality of the reduction can be easily quantified. The probability that *A* wins the $Game_{i}$ is denoted by $Pr[Success_{i}]$.

$\#Game_{1}:$*C* chooses a DAG *G* and picks a random homomorphic hash function *H* and a homomorphic pseudorandom generator PG and associates them with the compression vertices and expansion vertices of *G*, respectively. *C* then runs the key generation algorithm and provides the public key to *A*. Because *C* knows the entire secret key, it can easily provide any one-time signature on *A*’s requests. The success probability of this game is ϵ, so

(1) $$Pr[Success_{1}]=\epsilon$$

$Game_{2}:$ As $Game_{1}$, but with the modification that *C* picks an internal node at random and keeps it as the target node. *C* aborts if the adversary’s signature query includes an arc predeceasing the target node or if the adversary’s forgery does not invert the function on this node. Since the actions of *C* are independent of the chosen target, the forger behaves as in $Game_{1}$. The success probability is at least $\frac{Pr[Success_{1}]}{\left|v\right|}$. Hence, the success probability in $Game_{2}$ is

(2) $$Pr\left[Success_{2}\right]\leq \frac{\epsilon }{n}$$

$Game_{3}:$ As in $Game_{2}$, but with the following key generation change. *C* selects a target node on which to invert the function and replaces the selected node’s output value with *y*. *C* additionally assigns random values to all arcs leading out of the previous graph of the target node. *C* recalculates the public key and hands it on to *A*. Except for those within the predecessor graph of the target, *C* can generate a value for all arcs. The arcs directly going out of the predecessor nodes in a direct line of the target node are the only arcs for which *C* has no value. *C* responds to the signature question if the adversary’s inquiry does not contain any arcs within the preceding graph. *C* aborts the signature query if this is the case. When *A* wins $Game_{3}$, the adversary has assigned a value $y^{\prime }$ and its preimage to the targeted node. *C* discovered a preimage to the target if $y^{\prime }=y$. If $y^{\prime }\neq y$, on the other hand, a collision could occur somewhere along the way to the root. As a result, when *A* forges, $Game_{3}$ success results in either a collision on *H* or a preimage (for some $y_{H}$ or $y_{PG}$)

(3) $$Pr\left[Success_{3}\right]\leq Pr\left[Invert\;or\;Collision\right]\leq \epsilon_{PG}+\epsilon_{H}+\epsilon_{C}$$

It is also evident that, when *A* distinguishes between $Game_{2}$ and $Game_{3}$, it is distinguishing between a right evaluation of the graph consisting of the predecessors in a direct line of the goal, along with their offspring, and an equal number of uniformly chosen uniform random values. As a result, this chance is negligible if all of the predecessors are undetectable. When an efficient signature forger exists, then

(4) $$|Pr\left[Success_{3}\right]-Pr\left[Success_{2}\right]|\leq \alpha Pr\left[Detect\right]\leq \alpha \epsilon_{D}$$

From the sequence of the games and by considering the probability of finding collision on chameleon hash $\epsilon_{G}$, we have

(5) $$\epsilon \leq \left(\alpha \epsilon_{D}+\epsilon_{C}+\epsilon_{PG}+\epsilon_{H}\right)n+\epsilon_{G}$$

From Equation (5), if PG and *H* are cryptographically secure, then the HBCS scheme is unforgeable under a chosen message attack. □

**Theorem** **5.** *The proposed chameleon signature scheme satisfies the property of non-transferability.*

**Proof.**  The semantic security of the proposed hash scheme in Theorem 2 implies the non-transferability of the resulting chameleon signature scheme. □

**Theorem** **6.** *The proposed chameleon signature scheme satisfies the property of non-repudiation.*

**Proof.**  In the case of disputes, if a verifier provides a chameleon signature forgery, then the signer can repudiate it by producing collision pairs for the judge. By Theorem 1, the chameleon hash function is collision-resistant. Hence, in case of disputes, when the signer is not able to produce valid collision pairs on the chameleon hash to the judge, he is indeed the generator of the signature. □

**Theorem** **7.** *The proposed chameleon signature scheme satisfies the property of deniability.*

**Proof.**  It is ensured by the Denial protocol. □

## 7. Discussion

**Performance Analysis and Comparison:** The significant parameters for a graph-based signature scheme on a DAG *G* are the number of internal vertices, which reveals the number of function evaluations required to compute the public key from the secret key, the maximum number of incompatible signatures, which gives the upper bound on the size of the message space, and the maximal size of the signature.

The proposed scheme requires hash evaluations and pseudorandom evaluations for the computation of public key, signature generation, and the verification process. For a DAG with *n* internal vertices, the worst-case time complexity of hash evaluations and pseudorandom evaluations are $O(\frac{n}{2})$ and $O(\frac{nk}{2})$, respectively. When $n_{1}$ and $n_{2}$ are the number of vertices in $G_{S}$ and $G_{V}$, respectively, the time complexity of signature generation is $O\left(n_{1}k\right)+O\left(n_{2}k\right)$. The verification process also requires the same time.

For a given graph *G* with *n* internal vertices, let ν(n) be the width of the associated poset. Table 1 shows that, for a given *G* of *n* internal vertices, there are ν(n) compatible minimal allowable sets. By optimizing *n* and the structure of the graph, ν(n) can be increased and hence the scheme can achieve the best possible efficiency in terms of signature size and computational costs.

The size of the chameleon signature σ depends on the length of $r^{{}^{\prime }}$ and the length of λ. The length of λ depends on $n_{1}$—the number of internal vertices of $G_{S}$ and the length of $r^{{}^{\prime }}$ depends on the length of the output of the hash function *H* which is of *k* bits. The minimal allowable sets in the maximal antichain of the proposed graph construction in Algorithm 1 contain either $\frac{n_{1}}{2}$ or $\frac{n_{1}}{2}+1$ edges. Hence, λ consists of values of either $\frac{n_{1}}{2}$ edges or $\frac{n_{1}}{2}+1$ edges, where each value is of *k*-bits. Therefore, the maximum size of the signature $\sigma =\left(\frac{n_{1}}{2}+1\right)k+k=\left(\frac{n_{1}}{2}\right)k+2k$.

Few chameleon signature schemes are constructed by combining different chameleon hash functions proposed in [2,4,18] with the standard signature algorithms (RSA, DSS, etc.) of the same public key settings, and the comparison of those schemes with the proposed work for the security level of 80 bits is shown in Table 2—for instance, the hash function defined in [19] and the pseudorandom generator defined in the [19] proposed scheme. The hash function $H:{\left\{0,1\right\}}^{2k}\;\to \;{\left\{0,1\right\}}^{k}$ is defined by H(x)=Axmodq, where $A\in Z_{q}^{n\times 2k}$ and pseudorandom generator $PG:{\left\{0,1\right\}}^{k}\to {\left\{0,1\right\}}^{2k}$ is defined by $PG\left(x\right)=(g_{B}\left(s,x\right),g_{B}\left(s,x\right))$, where $g_{B}\left(s,x\right)\;=\;Bs\;+x\;mod\;q$ for $B\in Z_{q}^{n\times k}$ and a fixed secret $s\in Z_{q}^{k}$. In order to get 80-bit security, one needs to set the parameters of the functions *k*, *n* and *q* as k=nlogq and $q=n^{2}$ as suggested by Micciancio Daniele in [20]. To achieve comparable security, we set the discrete logarithm based schemes p=1024 and q=160 bits, and the RSA scheme’s RSA modulus to 1024 bits. The suggested quantum resistant system’s signature size is also compared to that of a quantum resistant lattice-based chameleon signature technique [13]. The parameters *q* = 100,000,007 and *n* equals the security level λ are used to determine the signature size, as indicated in [13]. The comparison results in Table 2 show that the signature length of the proposed scheme is larger than that of non-quantum schemes. This cost can be considered as the price for the proposed scheme to be quantum safe. In addition, the proposed scheme is key-exposure free and its signature length is smaller when compared to the existing quantum resistant chameleon signature scheme proposed in [13].

## 8. Conclusions

First, a new quantum-resistant hash-based chameleon hash technique with graph configuration is proposed in this study. It meets all of the desired security requirements, including collision resistance, semantic security, and key-exposure freeness. Second, utilizing the suggested chameleon hash and a graph-based one-time signature method, a privacy-preserving quantum-resistant chameleon signature technique is created. To the best of our knowledge, it is the first scheme under hash-based cryptography that could satisfy the quantum world’s requirement of privacy-preserving signatures. Furthermore, the comparison of the proposed scheme with the existing chameleon signature schemes shows that it has a smaller signature length than the existing quantum-safe scheme.

## Figures and Tables

**Table 1:** Table of *n* and ν(n).

*n*	ν(n)	*n*	ν(n)
4	2	18	66
6	5	20	86
8	8	22	117
10	14	24	147
12	20	26	190
14	33	28	232
16	45	30	289

**Table 2:** Comparison of the proposed HBCS scheme with existing chameleon signature schemes.

Scheme	System	Hard Problem	Signature Size (Bits)	KE	QS
Krawczyk et al. [2]	Num.-based	DLP	480	Yes	No
Ateniese & de Medeiros [18] + RSA	Num.-based	IFP	2048	Yes	No
Ateniese & de Medeiros [4] + DSS	Num.-based	DLP	640	No	No
Xie Dong et al. [13]	Lattice-based	SIS	$5\times 10^{9}$	No	Yes
Proposed HBCS	Hash-based	PR	$1\times 10^{4}$	No	Yes

Num: Number Theory, KE: Key Exposure, QS: Quantum Safe.

## Data Availability

Not applicable.
